# Evaluation of a Musculoskeletal Digital Assessment Routing Tool (DART): Crossover Noninferiority Randomized Pilot Trial

**DOI:** 10.2196/56715

**Published:** 2024-07-30

**Authors:** Cabella Lowe, Ruth Sephton, William Marsh, Dylan Morrissey

**Affiliations:** 1 Centre for Sports & Exercise Medicine William Harvey Research Institute Queen Mary University of London London United Kingdom; 2 St Helens Musculoskeletal Physiotherapy Service Mersey Care NHS Foundation Services St Helens United Kingdom; 3 Machine Intelligence and Decision Support [MInDS] Research Group School of Electronic Engineering and Computer Science and Digital Environment Research Institute Queen Mary University of London London United Kingdom; 4 Department of Physiotherapy Barts Health NHS Trust London United Kingdom

**Keywords:** mHealth, eHealth, digital health, digital technology, digital triage, musculoskeletal, triage, physiotherapy triage, validation, acceptability, physiotherapy, primary care, randomized controlled trial, usability, assess, assessment, triaging, referrals, crossover

## Abstract

**Background:**

Musculoskeletal conditions account for 16% of global disability, resulting in a negative effect on patients and increasing demand for health care use. Triage directing patients to appropriate level intervention improving health outcomes and efficiency has been prioritized. We developed a musculoskeletal digital assessment routing tool (DART) mobile health (mHealth) system, which requires evaluation prior to implementation. Such innovations are rarely rigorously tested in clinical trials—considered the gold standard for evaluating safety and efficacy. This pilot study is a precursor to a trial assessing DART performance with a physiotherapist-led triage assessment.

**Objective:**

The study aims to evaluate trial design, assess procedures, and collect exploratory data to establish the feasibility of delivering an adequately powered, definitive randomized trial, assessing DART safety and efficacy in an NHS primary care setting.

**Methods:**

A crossover, noninferiority pilot trial using an integrated knowledge translation approach within a National Health Service England primary care setting. Participants were patients seeking assessment for a musculoskeletal condition, completing a DART assessment and the history-taking element of a face-to-face physiotherapist-led triage in a randomized order. The primary outcome was agreement between DART and physiotherapist triage recommendation. Data allowed analysis of participant recruitment and retention, randomization, blinding, study burden, and potential barriers to intervention delivery. Participant satisfaction was measured using the System Usability Scale.

**Results:**

Over 8 weeks, 129 patients were invited to participate. Of these, 92% (119/129) proceeded to eligibility assessment, with 60% (78/129) meeting the inclusion criteria and being randomized into each intervention arm (39/39). There were no dropouts and data were analyzed for all 78 participants. Agreement between physiotherapist and DART across all participants and all primary triage outcomes was 41% (32/78; 95% CI 22-45), intraclass correlation coefficient 0.37 (95% CI 0.16-0.55), indicating that the reliability of DART was poor to moderate. Feedback from the clinical service team led to an adjusted analysis yielding of 78% (61/78; 95% CI 47-78) and an intraclass correlation coefficient of 0.57 (95% CI 0.40-0.70). Participant satisfaction was measured quantitively using amalgamated System Usability Scale scores (n=78; mean score 84.0; 90% CI +2.94 to –2.94), equating to an “excellent” system. There were no study incidents, and the trial burden was acceptable.

**Conclusions:**

Physiotherapist-DART agreement of 78%, with no adverse triage decisions and high patient satisfaction, was sufficient to conclude DART had the potential to improve the musculoskeletal pathway. Study validity was enhanced by the recruitment of real-world patients and using an integrated knowledge translation approach. Completion of a context-specific consensus process is recommended to provide definitive definitions of safety criteria, range of appropriateness, noninferiority margin, and sample size. This pilot demonstrated an adequately powered definitive trial is feasible, which would provide evidence of DART safety and efficacy, ultimately informing potential for DART implementation.

**Trial Registration:**

ClinicalTrials.gov NCT04904029; http://clinicaltrials.gov/ct2/show/NCT04904029

**International Registered Report Identifier (IRRID):**

RR2-10.2196/31541

## Introduction

### Background

Musculoskeletal conditions are a global epidemic, prevalent across all ages and increasing rapidly [1-3], being associated with increased life expectancy and reduced activity [4,5]. In the United Kingdom, musculoskeletal conditions pose a financial and societal challenge, costing over £4.76 billion (US $5.99 billion) of the UK National Health Service (NHS) resources and using up to 1 in 3 primary care physician visits annually [6,7]. Patients use more health care and generate higher costs if they must wait longer for assessment and treatment [8,9] with longer waiting times potentially leading to detrimental effects on pain, disability, and quality of life for waiting patients [8,9], as well as increasing their risk of chronic health disease [10]. “Getting It Right First Time” by directing patients to the correct level of intervention at the first point of contact, is considered key in improving condition outcomes and reducing unwarranted variation in clinical pathways, such as unnecessary secondary care consultations and investigations [11].

Remote physiotherapist-led musculoskeletal triage services are widely used within the NHS and private sectors and have the potential to reduce waiting times, musculoskeletal caseload, and cost across the pathway [12-14]. However, the principal rate-limiting factor on the ability of services to increase activity and treat more patients is the availability of staff [15].

It has been suggested that mHealth technology could provide a cost-effective alternative to physiotherapist-led remote triage for improving health care delivery [16,17], with recent advances being made in digital primary care triage applications [18,19]. Using a digital triage tool has the potential to screen for conditions requiring emergency or urgent care, while directing less complex or urgent presentations to routine physiotherapy or supported self-management, thereby maximizing the use of highly skilled clinicians’ time.

However, a web-based triage platform directing patients with musculoskeletal presentations to an appropriate level of care requires robust testing and validation prior to implementation [20-28]. To date there is limited evidence regarding the use of web-based or digital triage platforms for musculoskeletal conditions specifically, with most investigations focused on the performance of generic symptom checkers covering a wide range of clinical presentations. Evidence from these studies concerning clinical and cost-effectiveness, signposting to appropriate services, patient compliance, and safety was found to be weak or inconsistent [16,29] and most notably, not conducted in a setting relevant to the UK health and social care system. The methodological challenges documented by other digital intervention researchers could, in part, contribute to the validity of results [30] and we sought to draw on their experience to improve the validity of our main trial results by testing our system in a real-world setting. A randomized controlled trial (RCT) is considered the gold standard methodological design to reduce conscious or unconscious bias, using randomization and blinding to ensure no false conclusions are drawn from the study research [31], with piloting required to ensure trial success. For our study, we chose a noninferiority design, not determining if triage performed using a digital assessment routing tool (DART) was superior to physiotherapist-led triage, rather if it was not “unacceptably worse” [32]. This allowed consideration of potential nonclinical benefits such as patient convenience, satisfaction, and cost-effectiveness. The pilot study described in this paper was to ensure the successful delivery of a main trial examining DART safety and efficacy and to assess the suitability of the RCT design for evaluating a digital triage system.

### DART Overview

**Figure 1:**
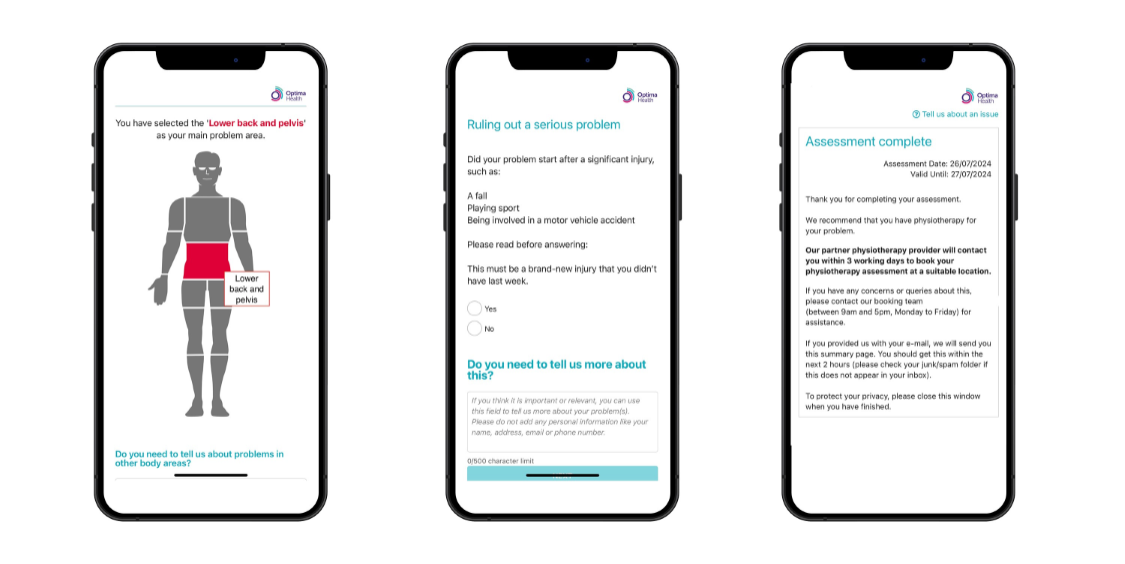
Digital assessment routing tool mobile health system user display examples.

DART (developed by Optima Health) is a web-based first-contact mHealth system designed specifically to direct patients with musculoskeletal disorders to the correct level of care (Figure 1). DART contains an algorithm driving question and response options leading to a triage recommendation configured to match the provider’s clinical services, based on evidence-based practice, clinical guidelines, and sector-specific referral criteria. For this reason, there may be variants of DART, containing subtle differences to ensure the algorithm is mapped to the musculoskeletal service in which it sits. Triage recommendation options may include emergency or routine medical assessment, physiotherapy, self-management programs, or psychological support services. For this study, the DART algorithm was mapped to the specific NHS musculoskeletal service delivered at the trial site. DART is a web app, only accessible by users via the musculoskeletal service provider’s website. It is not intended for general population use via the app store. DART is classified as a “symptom checker” by the UK Medicines and Healthcare Products Regulatory Agency and so does not qualify as a medical device [25]. It is classified as a tier C system by the UK National Institute of Health and Care Excellence whose classification groups align with those proposed by the International Medical Device Regulators Forum [20]. It has been used within a controlled real-world occupational health setting within Optima Health since 2019 with over 9000 assessments being completed.

### Previous Work

Previous work as described in the pilot protocol [33] included an assessment of clinical validity by an expert panel, real-world usability testing [34], and assessment within a controlled clinical environment.

### Aims and Objectives

In this pilot trial, the research aim was to evaluate trial design, assess procedures, and collect exploratory data to assess the feasibility of delivering an adequately powered, definitive crossover noninferiority randomized trial, assessing DART safety and efficacy in an NHS primary care setting.

The primary objective of the trial was to collect and synthesize data (agreement of triage outcome made between DART and physiotherapist-led triage) to define a noninferiority margin and subsequent sample size calculation for an adequately powered main trial using the principles described by Bujang and Baharum [35]. Agreement was defined as the physiotherapist selecting the same triage recommendation as given by DART (Textbox 1).

**All possible triage outcomes and suboutcomes from digital assessment routing tool and physiotherapist-led triage assessment.**

**Medical care**

- Emergency care (emergency department referral)
- Urgent primary care physician (general practitioner [GP])
- Routine primary care physician (GP)
- Consultant review

**First contact practitioner (FCP) physiotherapist**

- Urgent FCP
- Routine FCP

**Physiotherapy care**

- Postfracture or surgery physiotherapy
- Physiotherapy referral
- Physiotherapy referral plus psychosocial support

**Remote self-management**

- Supported self-management
- Web-based support material

Secondary process objectives were as follows with associated predefined outcomes:

- Recruitment (recruitment rate targets=50%, retention=95%, and dropouts<4)
- Randomization (equal numbers allocated to each intervention arm, occurrences of allocation concealment failure, and introduction of bias)
- Effectiveness of process implementation (occurrence of nonadherence to study protocol, DART login errors, and DART system failures)
- Burden on patients and clinician (measurement of treatment delays and additional time requirements and feedback from physiotherapists and researchers concerning trial procedure complexity).
- Participant satisfaction with using DART (amalgamated System Usability Scale [SUS] scores), with the expectation that a mean score of 80 or more would be achieved, a standard consistent with the previously published DART usability study [34].

## Methods

### Study Design

This 8-week crossover noninferiority randomized controlled pilot trial was conducted within an NHS primary care setting, using equal randomization of 1:1. The study was designed in accordance with the CONSORT (Consolidated Standards of Reporting Trials) guidelines for pilot and feasibility trial [36], CONSORT guidelines for equivalence and noninferiority randomized trials [37] and EHEALTH checklist [38]. While the terms feasibility and pilot are often used interchangeably, the term “pilot” trial was chosen by the authors to reflect that the methodology used would be reproduced in a future definitive RCT [36].

All participants underwent a web-based DART assessment and a face-to-face assessment with the on-site physiotherapist. The physiotherapist assessment was intended to reproduce the type of questioning a triage physiotherapist would deliver remotely over the telephone, providing a source of “ground truth” with which to compare the DART outcome, in fact potentially providing greater rigor by virtue of the physiotherapist being able to observe and interact with the patient. The physiotherapist assessment consisted of patient history taking and discussion of symptoms but did not include a physical examination. Only the triage outcome from this element was used for study comparison.

An integrated knowledge translation approach as described by Smith et al [39] was adopted, where the musculoskeletal services’ leader, lead primary care physician, and study physiotherapist all helped to shape the research, with the aim of improving its use and impact. This included discussions of triage routing to improve the alignment of the DART algorithm alignment with that of the existing clinical service prior to commencing the pilot. A minimum sample size of 76 participants was chosen based on the estimated stepped rules of thumb from Whitehead et al [40] to demonstrate an extra small, standardized effect size (SD <0.1) at a 90% powered main trial.

### Trial Setting

The Haydock Medical Centre is a well-established multidisciplinary primary care practice in the Northwest of England, with 50 staff and clinicians, serving over 15,000 patients. Through links with Health Education Northwest, Manchester, and Edge Hill Universities, it provides training for primary care physicians, medical students, nurses, and health care assistants. The more recent introduction in the United Kingdom of musculoskeletal first contact practitioner (FCP) physiotherapists located within primary care clinics is seen as providing an effective alternative to primary care physician or general practitioner (GP) assessment for musculoskeletal conditions, so potentially freeing up physician appointments [41]. The FCP physiotherapist who participated in this trial was based 2 days per week at the center and patients presenting with musculoskeletal symptoms were either booked directly into the FCP physiotherapist diary instead of seeing a primary care physician or were referred to the FCP physiotherapist by another clinician at the practice. By virtue of enhanced clinical skills beyond that of most triage physiotherapists, an FCP physiotherapist is trained to manage more complex cases and may facilitate diagnostic investigation and refer to specialist services. For this reason, the on-site FCP physiotherapist was chosen to provide the subjective physiotherapist assessment, to act as a rigorous study comparator with which to evaluate DART.

### Recruitment

DART has been designed to triage patients self-referring into primary care for any suspected musculoskeletal condition, and therefore is not limited to any specific type or stage of injury. Patients may also be directed by their primary care physician to use DART to confirm the type of musculoskeletal care required. Posters and leaflets advertising the study were placed in the practice waiting room. Patients with a musculoskeletal condition wishing to access support from the practice (either primary care physician or FCP), were offered the opportunity to participate in the study by the reception team at the point of requesting an appointment, either in person at the practice or by telephone. Patients were provided a brief eligibility screen and a short description of the study, with those wishing to participate subsequently booked into a 45-minute slot in the trial diary. This appointment duration allowed both assessments to take place in addition to obtaining informed consent, randomization, and blinding processes.

### Inclusion and Exclusion Criteria

The study participant inclusion criteria were as follows: (1) adults aged greater than 18 years; (2) able to speak and read English; (3) registered patient at the primary care practice; (4) current musculoskeletal condition for which they were seeking treatment; and (5) able to access the internet either themselves or with the help of family or friend.

The study participant exclusion criteria were as follows: (1) significant physical or cognitive impairments sufficient to limit their ability to follow study-related procedures; (2) unwillingness to follow protocol-related procedures; (3) an existing diagnosis for their condition given by a medical professional within the last 7 days; and (4) Optima Health employees. Participants were not paid to participate in the trial.

### Ethical Considerations

This study received human subjects research ethical approval from the Health Research Authority, London-Surrey Borders Research Ethics Committee on March 24, 2022 (22/LO/0129).

To support informed consent, on arrival participants were given the participant information sheet (Multimedia Appendix 1) outlining the purpose of the trial and the nature of their participation. This included information about the format of the interaction, potential risks, confidentiality and protection of their personal data, use of their data for analysis (including secondary analysis by expert panel review), anonymity of study findings, and their right to withdraw at any time without prejudice. In addition, this document signposted patients to the Queen Mary University of London Privacy Notice (Multimedia Appendix 2). Patients were made aware no remuneration was to be given for participation. They were then given the opportunity to raise questions with the researcher during the formal consenting process, which was conducted in an allocated treatment room. Formal informed consent was obtained by the researcher and documented using a web-based form (Multimedia Appendix 3). Failure to provide consent resulted in the patient immediately receiving a usual care assessment from the on-site physiotherapist, as per the trial protocol [33].

### Data Collection

The sequence of assessments was determined by randomization to account for order effects in the crossover design and achieved by block randomization with permuted blocks of random size and without stratification factors to avoid selection bias and unequal arms [42-44]. After gaining consent, the researcher used Sealed Envelope software [45] to generate a randomization sequence with a 1:1 allocation ratio between the study arms. Triage outcomes within DART were matched to those available to the physiotherapist based on usual care approaches in musculoskeletal clinical practice. These outcomes were classified into 4 categories: medical care, FCP (referral for assessment with an FCP), physiotherapy care, and remote self-management. There were further suboutcomes within each category including levels of urgency (Textbox 1) allowing direct comparison between the 2 types of the triage assessment outcome. Levels of care were determined by the clinician’s skill set and their access to diagnostic or treatment facilities, with medical care able to provide the greatest level of support and remote self-management the least.

The physiotherapist assessment was completed within a 20-minute appointment, which included standardization of time for each study arm to support blinding. To minimize potential bias, the physiotherapist did not discuss any possible diagnosis or give condition management advice to the participant until their assessment had been completed and their study outcome documented. The DART web-based assessment was completed in a clinic room adjacent to the physiotherapist’s room, either before or after the appointment with the physiotherapist, depending on the randomization allocation. The researcher logged participants onto DART using a tablet device and explained they would not be able to assist or discuss any of the questions with them. If the participant said they normally used the internet with help from a family member or friend as a surrogate seeker [46], the researcher would assist in this way to navigate through the DART assessment and read the text but would not discuss clinical details at any stage with the participant. The participant followed the instructions given by DART until they arrived at the final page where the DART outcome was not visible to either participant or researcher but stored in DART for later retrieval and analysis. Thereafter, the participant completed the web-based SUS questionnaire to measure user satisfaction with DART [47]. Both assessments were completed at the same visit and within 10 minutes of each other to reduce variation in clinical presentation. Once the participant had finished both study assessments and data collection complete, they returned to the physiotherapist, who performed a physical assessment and continued with normal care. Participants could opt out of the study at any point, which did not affect their usual physiotherapist-led management. This process was supported through documented guidance and training delivered by the principal investigator. Blinding was ensured at three different points: (1) the physiotherapist was blinded to group allocation and DART assessment outcomes, (2) participants were blinded to the DART assessment outcome and the physiotherapist triage outcome until they have completed both assessments and the SUS, and (3) the analysis and interpretation of the study results was completed by researchers blinded to the intervention group allocation.

### Data Analysis

An independent panel consisting of 3 experts qualified to consultant level in musculoskeletal physiotherapy and general practice, provided consensus on all disagreements between DART and physiotherapy-led triage that would yield a safety concern, which were as follows:

- When the DART outcome was physiotherapy or self-management, and the physiotherapist outcome was emergency or urgent medical care (emergency department referral or urgent primary care physician)
- When the DART outcome was self-management and the physiotherapist outcome was physiotherapy, FCP, or medical care
- When the DART outcome was routine care, and the physiotherapist outcome was emergency or urgent care.

In addition, a random sample of 10% of the remaining cases that did not yield a safety concern were assessed by the panel to decide what they considered to be the correct outcome. This was based on the participant’s presentation from the physiotherapist’s assessment clinical record and the DART assessment summary which provided the questions asked and the participant’s responses. Where 2 or more panel members disagreed with the physiotherapist’s decision, the panel recommendation was used to provide the definitive outcome against which the DART outcome was compared. In all cases where DART did not agree with the physiotherapist outcome further analysis was performed to ascertain the direction and extent of escalation. Underescalations (where DART recommended a lower level of clinical support than the physiotherapist) and overescalations (where DART recommended a higher level of clinical support) were assigned to levels 1, 2, or 3, depending on the difference in number of levels between physiotherapist and DART outcomes. Data collected from DART, physiotherapist, web-based SUS questionnaire, and researcher log were entered on to an Excel spreadsheet by the principal investigator and checked for accuracy by a second researcher prior to analysis. Qualitative data from the physiotherapist, NHS service lead, and researchers regarding the study process was noted during informal poststudy debrief discussion sessions.

### Statistical Analysis

The primary analysis was an absolute agreement intraclass correlation coefficient (ICC; A,2) estimate with 95% CIs between DART and the physiotherapist across all triage outcomes, with a subanalysis of categories (medical referral, FCP, physiotherapy, and self-management) and adverse triage outcomes. This was calculated using SPSS statistical package software (version 23; SPSS Inc) and based on a single rating, 2-way random-effects model [48,49]. The ICC was reported with a 95% CI which gave a measure of reliability as described by Koo and Li [48]. This measure of agreement would inform a consensus for the noninferiority margin required for the main study, in turn facilitating a definitive trial sample size calculation. DART user satisfaction scores were reported as a mean SUS score and adjective rating across all participants.

## Results

### Recruitment

**Figure 2:**
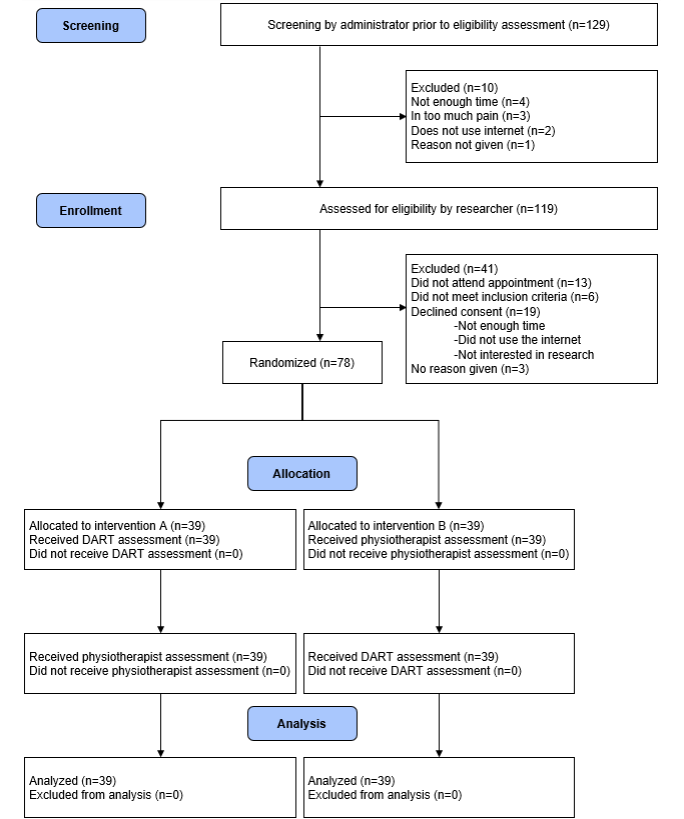
CONSORT (Consolidated Standards of Reporting Trials) diagram showing flow of participants through the study. DART: digital assessment routing tool.

A total of 129 patients contacted the practice seeking an appointment for a suspected musculoskeletal condition during the 8-week trial period, with 92% (119/129) passing initial eligibility screening and being booked into the study (Figure 2). Of these, 35% (41/119) were excluded by the researcher owing to the following reasons: 13 not attending their appointments, 6 not meeting the inclusion criteria, and 19 declining consents (with the most common reasons given as not having enough time, did not use the internet and not interested in research). A further 3 patients were unable to participate due to technical issues related to internet connectivity. Recruitment continued until the predefined sample size of 76 had been exceeded. A total of 60% (78/129) of participants were enrolled in the study. This exceeded the predefined recruitment rate of 50%. There were no dropouts and 100% retention of participants, exceeding the predefined level of 95%. All data were collected during the single appointment, with no missing data.

### Randomization and Blinding

**Figure 3:**
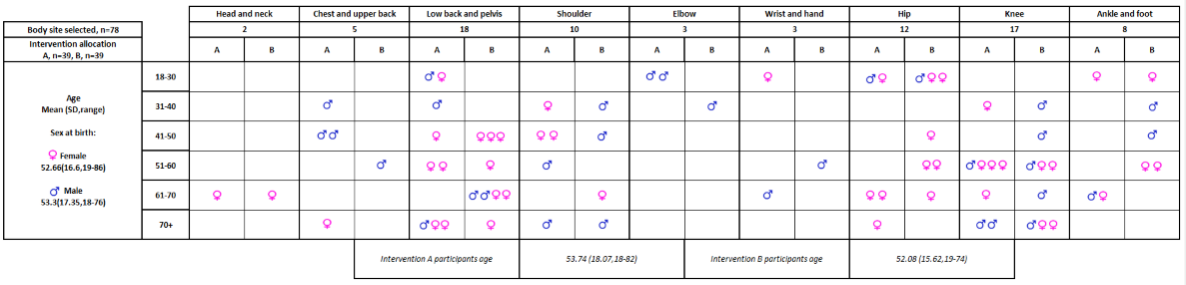
Baseline participant demographic characteristics for all participants (n=78) by body site, randomized intervention allocation, sex at birth, and age.

Randomization was effective with participants evenly distributed across the 2 intervention arms (A=39 and B=39) and no failures of allocation concealment. The 2 trial interventions arms were evenly matched in terms of sex at birth and age, with homogeneity, indicative of successful randomization and minimized risk of selection bias (Figure 3). Bias was minimized through standard timings for both types of assessment, however, this meant patients arriving more than 10 minutes late for their appointment were unable to participate, with 3 participants excluded for this reason. Researchers noted participants often wished to engage in discussion about their musculoskeletal condition while waiting for their physiotherapist assessment and suggested researchers should leave the room except when performing study-related activities to minimize this risk.

### Data Collection

Process implementation was effective with full adherence to the trial protocol, evidence of which was documented for each participant on the researcher log. There were 3 patients who would have been eligible to take part; however, lack of internet connection within the study area meant they could not participate. Otherwise, there were no DART login errors or DART system failures during data collection. The burden on patients and clinicians was considered acceptable, as there were no treatment delays beyond the 15 minutes taken to complete the study process and participants had no extra travel in addition to that required for their physiotherapy appointment. There was no harm to any participants or unintended effects or consequences. Researchers said the data collection was procedurally complex for them to deliver, particularly around the accuracy of timings to maintain blinding; however, the physiotherapist reported their part in the process was straightforward. The additional diary time allocated to the physiotherapist to complete the trial process, over and greater usual care was 20 minutes per participant, and for the researcher 45 minutes per participant, the cost of which would need to be factored into the delivery of a future definitive trial.

### Protocol Deviations

During the trial, the study physiotherapist identified challenges in making decisions for the FCP primary outcome due to the ambiguity of the FCP referral definition within the protocol. After discussion between the principal investigator and physiotherapist, it was decided to continue as per the study protocol, but once data collection was complete the physiotherapist would review all 22 cases previously routed to FCP and either confirm or amend their outcome prior to data analysis based on clarification of the FCP referral criteria. The demographic characteristics of participants are presented in Figure 3. More females were recruited than males (60%:40%), a ratio higher than reported UK musculoskeletal prevalence [7]. The mean age of all participants was 52.9 (SD 16.79) years with a range from 18 to 78 years.

As shown in Figure 3, the most frequently seen age group was 46-65 years (31/78, 40%), with the 65 and greater group representing 27% (21/78). The prevalence of musculoskeletal conditions is reported as increasing with age [7], so it is likely that older participants are underrepresented in this study. The most frequently selected body sites were lower back and pelvis, 23% (18/78), and knee, 22% (17/78) consistent with a recent study examining musculoskeletal presentations within a similar urban community primary care practice [50]. Hip conditions represented the next most frequently selected site with 15% (12/78). These 3 body sites accounted for 60% (47/78) of all presentations.

### Panel Review

The panel of 3 experts reviewed 14 cases (Table 1). The protocol requirement for a random sample of 10% of participants in addition to the safety cases was exceeded by 1 case due to researcher error. There was complete agreement between all panel members for 57% (8/14) cases, and partial agreement between 2 panel members for the remaining 43% (6/14) cases. As per the study protocol, where 2 panel members agreed on the same outcome that differed from that of the physiotherapist, the panel outcome was used for data analysis. This resulted in 3 changes to the physiotherapist’s outcome, all of which were the same as the DART outcome. There were 5 cases where 1 panel member disagreed with the physiotherapist’s outcome but was insufficient to trigger a change.

The updated physiotherapist outcomes were used in the primary outcome data analysis (Table 2).

**Table 1:** Primary outcome by digital assessment routing tool (DART), study physiotherapist, and expert panel members. Some DART outcomes are classified as adverse (n=5) and the remainder represents a further randomly selected 10% sample (n=9) of participants, plus 1 additional selected in error. Highlighted physiotherapist outcomes signify a panel change, with the revised outcome shown in brackets. Revised outcomes were used for data analysis (n=3).

DART^a^	Physiotherapist	Expert 1	Expert 2	Expert 3
Self-management^b^	Physiotherapy	Physiotherapy	Physiotherapy	Physiotherapy
Self-management	Self-management	Self-management	Self-management	Self-management
Self-management	FCP^c^ (self-management)	Self-management	Self-management	Self-management
Medical	FCP (medical)	Medical	Medical	Medical
Physiotherapy^b^	FCP	FCP	FCP	Physiotherapy
Self-management	Self-management	Self-management	Self-management	Self-management
Self-management	Self-management	Self-management	Self-management	Self-management
Self-management^b^	FCP	FCP	FCP	FCP
Physiotherapy	Physiotherapy	Physiotherapy	Physiotherapy	Physiotherapy
Physiotherapy	Physiotherapy	Physiotherapy	Physiotherapy	FCP
Physiotherapy	FCP (Physiotherapy)	FCP	Physiotherapy	Physiotherapy
Self-management^b^	Physiotherapy	Physiotherapy	Self-management	Physiotherapy
Self-management^b^	Physiotherapy	Physiotherapy	Physiotherapy	Self-management
Medical	FCP	FCP	FCP	Medical

^a^DART: digital assessment routing tool.

^b^Dart outcomes classified as adverse.

^c^FCP: first contact practitioner.

**Table 2:** Physiotherapist and digital assessment routing tool primary and secondary outcomes for all participants. Adverse outcomes are shaded.

Agree or escalation (primary outcome)	Escalation level	Physio primary outcome	DART^a^ primary outcome	Physio secondary outcome	DART secondary outcome	Cases
Agree	N/A^b^	Medical	Medical	Routine primary care physician (GP^c^)	Routine primary care physician (GP)	1
Agree	N/A	FCP^d^	FCP	Routine FCP	Routine FCP	1
Agree	N/A	FCP	FCP	Routine FCP	Urgent FCP	1
Agree	N/A	Physiotherapy	Physiotherapy	Physiotherapy referral	Physiotherapy referral	14
Agree	N/A	Physiotherapy	Physiotherapy	Physiotherapy+psychosocial support	Physiotherapy referral	2
Agree	N/A	Physiotherapy	Physiotherapy	Physiotherapy referral	Physiotherapy+psychosocial support	1
Agree	N/A	Self-management	Self-management	Supported self-management	Supported self-management	2
Agree	N/A	Self-management	Self-management	Supported self-management	Web-based support material	2
Agree	N/A	Self-management	Self-management	Web-based support material	Web-based support material	7
Agree	N/A	Self-management	Self-management	Web-based support material	Supported self-management	2
Underescalation	Level 1	FCP	Physiotherapy	Routine FCP	Physiotherapy referral	3
Underescalation	Level 1	Physiotherapy	Self-management	Physiotherapy referral	Supported self-management	1
Underescalation	Level 1	Physiotherapy	Self-management	Physiotherapy referral	Web-based support material	3
Underescalation	Level 2	Medical	Physiotherapy	Routine primary care physician (GP)	Physiotherapy referral	2
Underescalation	Level 2	FCP	Self-management	Routine FCP	Web-based support material	1
Overescalation	Level 1	FCP	Medical	Urgent FCP	Emergency care	2
Overescalation	Level 1	FCP	Medical	Routine FCP	Emergency care	2
Overescalation	Level 1	FCP	Medical	Routine FCP	Urgent primary care physician (GP)	1
Overescalation	Level 1	Physiotherapy	FCP	Physiotherapy +psychosocial support	Urgent FCP	2
Overescalation	Level 1	Physiotherapy	FCP	Physiotherapy referral	Routine FCP	2
Overescalation	Level 1	Self-management	Physiotherapy	Supported self-management	Physiotherapy referral	7
Overescalation	Level 1	Self-management	Physiotherapy	Web-based support material	Physiotherapy+psychosocial support	1
Overescalation	Level 1	Self-management	Physiotherapy	Web-based support material	Physiotherapy referral	8
Overescalation	Level 2	Physiotherapy	Medical	Physiotherapy referral	Emergency care	4
Overescalation	Level 2	Physiotherapy	Medical	Physiotherapy referral	Urgent primary care physician (GP)	1
Overescalation	Level 2	Physiotherapy	Medical	Physiotherapy referral	Routine primary care physician (GP)	1
Overescalation	Level 2	Self-management	FCP	Web-based support material	Urgent FCP	3
Overescalation	Level 3	Self-management	Medical	Supported self-management	Emergency care	1

^a^DART: digital assessment routing tool.

^b^N/A: not applicable.

^c^GP: general practitioner.

^d^FCP: first contact practitioner.

### Primary Objective

Following the adjustments made by the expert panel, the agreement between physiotherapist and DART across all participants and all primary outcomes was 33/78 (42%; 95% CI 22-45), an ICC of 0.37 (95% CI 0.16-0.55), indicating that the reliability of DART was poor to moderate [48]. Analysis of cases where there was an agreement between the physiotherapist and DART by the primary outcome is shown in Table 3.

**Table 3:** Agreement between physiotherapist and digital assessment routing tool by primary outcomes for all participants.

Primary stratification outcome	Rate	% (95% CI)
Medical	1/3	33 (0-6)
FCP^a^	2/11	18 (0-7)
Physiotherapy	17/31	55 (10-27)
Self-management	13/33	39 (7-27)

^a^FCP: first contact practitioner.

There were just 3 medical outcomes selected by the physiotherapist, none were emergency or urgent care, all being routine primary physician, DART agreed in 1 out of 3 cases. The greatest agreement at 55% was for the physiotherapy outcome and the lowest was for the FCP outcome, with DART agreeing with only 2 out of 11 FCP cases presented.

There were 5 cases meeting the protocol criteria of an adverse outcome representing a potential clinical safety issue: physiotherapy or self-management when it should have been emergency or urgent medical care (n=0), self-management when it should have been either physiotherapy, FCP, or medical care (n=5) and routine care when it should have been urgent care (n=0). In 4 out of 5 of these adverse outcomes DART underescalated by 1 level to self-management when the physiotherapist had routed to physiotherapy. The remaining case was an underescalation by DART to self-management when the physiotherapist had routed to FCP. During data analysis, it became clear this was created by a foot complaint screening question which has subsequently been revised.

**Figure 4:**
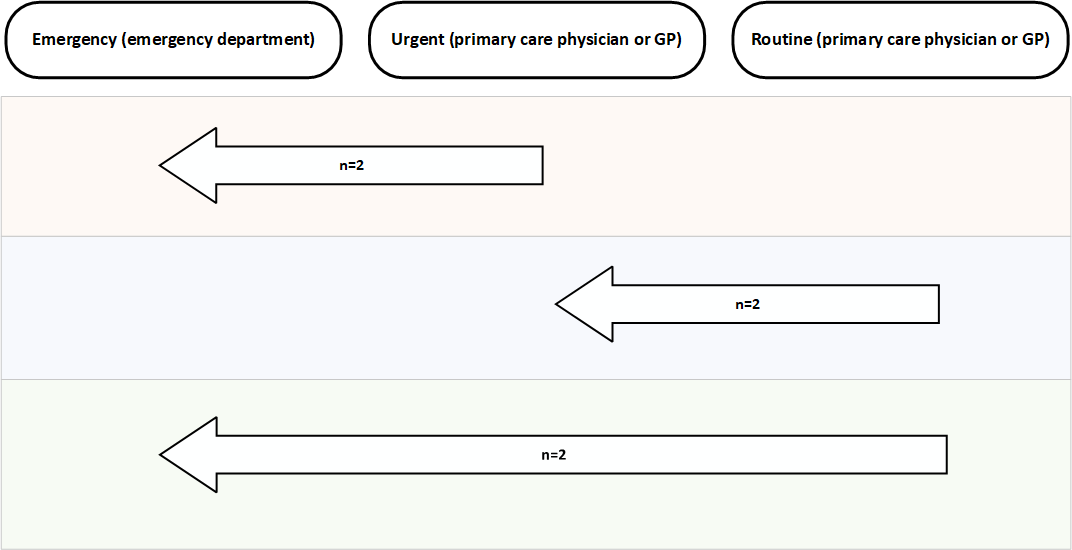
Number of DART overescalations by urgency of outcome (n=6). DART escalated 6 cases to urgent care where the physiotherapist had recommended routine care. The base of arrow indicates physiotherapist stratification outcome; head of arrow indicates DART outcome. DART: digital assessment routing tool; GP: general practitioner.

Urgency of referral was defined as secondary outcomes within the medical and FCP primary outcomes: emergency (emergency department), urgent (primary care physician [GP] or FCP and routine (primary care physician [GP] or FCP). Physiotherapy and self-management were considered routine in terms of urgency. DART overescalated 6 cases from routine to urgent (Figure 4). There were no cases where DART underestimated the urgency of the recommendation; however, no participants were deemed by the physiotherapist to require emergency or urgent medical care; therefore DART routing was not assessed in this area.

**Figure 5:**
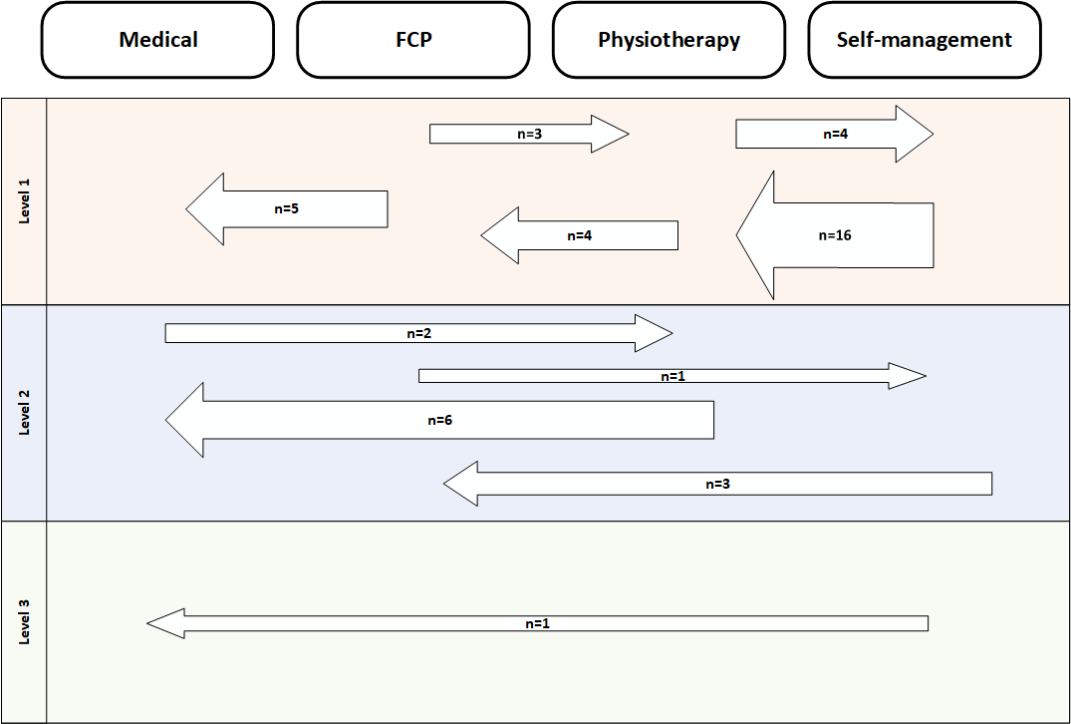
Breakdown of all physiotherapist-DART routing disagreements (n=45). Arrows pointing to the right indicate underescalations, those pointing to the left indicate overescalations. Base of arrow indicates physiotherapist outcome, head of arrow indicated DART outcome. Size of arrows are indicative of volume of cases. DART: digital assessment routing tool; FCP: first contact practitioner.

All DART under and overescalations are shown in Figure 5. A total of 10 cases were underescalated and recommended by DART to a lower level of intervention than that given by the physiotherapist. Of these, the majority were level 1 underescalations (7/10), with the remainder being level 2. DART overescalated the outcome for 45% (35/78) of all cases across all possible primary outcomes. Of these, 71% (25/35) were level 1 overescalations. Most overescalations occurred when the physiotherapist recommended self-management and DART gave an outcome of physiotherapy, accounting for 46% (16/35) of overescalations. There was only 1 case where escalation was 3 levels, where the body site selected was knee and the DART recommendation was medical instead of self-management. On further analysis, the participant’s response to a serious pathology screening question may have triggered a false positive outcome.

Statistical analysis of secondary outcomes when there was agreement on primary outcome was not performed; however, it was noted there was secondary outcome agreement between the physiotherapist and DART in 76% (25/33) cases.

Satisfaction using DART was measured quantitively across all participants using amalgamated SUS scores (n=78; mean score 84.0; 90% CI +2.94 to –2.94). A satisfaction adjective was associated with each participant’s individual total score to aid in explaining results to nonhuman factor professionals [51], with 74 out of 78 participant scores equating to DART as a “good,” “excellent,” or “best imaginable system.” The final mean SUS score of 84.0 achieved the predefined objective of a score of 80 or greater, representing a “good” or better system, associated with an increase in probability that users would recommend DART to a friend [52]. This score is consistent with that derived from the previous DART usability study of 84.3 [34]. Using the normalizing process described by Sauro [53] this ranked DART within the 96-100 percentile (SUS score 84.1-100) of systems tested using the SUS with an associated adjective rating of “excellent” [51]. Benchmarking of the DART SUS score against the mean score of 67 (SD 13.4; 90% CI) from 174 studies assessing the usability of public-facing websites using the SUS, revealed that DART was among the highest scoring systems assessed in this way [53,54].

### Adjusted Analysis

Following the collaborative approach taken within this trial, feedback was obtained from the expert panel and results were shared with the study physiotherapist and the NHS physiotherapy service clinicians to assess the strengths and weaknesses of the pilot design and identify areas for improvement prior to the definitive trial. It was noted that while the primary outcome of the pilot was not intended as a measure of DART safety and efficacy, the level of agreement between the study physiotherapist and DART outcome was lower than anticipated. A total of 3 key areas were identified to have influenced the primary outcome, and the following amendments to the study protocol were suggested for the future trial. First, the study physiotherapist and the expert panel highlighted the challenge posed by “borderline” cases, where participants could have arguably been correctly recommended for more than 1 primary outcome without compromising patient safety, particularly between physiotherapy and self-management. The concept of “acceptable differences” by using clinical judgment has been described previously, Bland and Altman [55] and could be applied in this context. The introduction of an “arguably correct” option for the panel to select when determining the level of physiotherapist-DART agreement would allow for the variability inherent between individual patient presentations and clinical reasoning. Second, it was noted that 4 of 5 cases defined by the protocol as adverse incidents were when the study physiotherapist recommended physiotherapy, while DART directed to self-management. The clinical team concluded there was no significant clinical risk if safety netting information was provided by DART encouraging patients to attempt self-management initially and direct them to physiotherapy via patient-initiated follow-up if unsuccessful. Safety netting can be described as a method of managing diagnostic uncertainty by providing information to patients and legitimizing a follow-up appointment, to ensure patients do not “slip through the net” [56]. This would replicate normal practice within the existing musculoskeletal service. Therefore, this underescalation between physiotherapy and self-Management should be included as an acceptable level of agreement within the study protocol. However, other types of level 1 underescalations should remain clinically unacceptable due to the potential for serious symptoms requiring an FCP or medical review. Third, the level 1 DART overescalations were considered in the context of managing clinical risk, acknowledging that neither digital health technology nor clinicians would agree all the time. It was concluded this level of false positive overescalation was acceptable and preferable to the increased risk of false negatives being generated by DART, consistent with a risk-adverse view taken by other developers of digital health technologies [57]. It was suggested that in a real-world musculoskeletal pathway, overescalations could be routed for a priority remote consultation with a physiotherapist to validate an onward referral.

**Figure 6:**
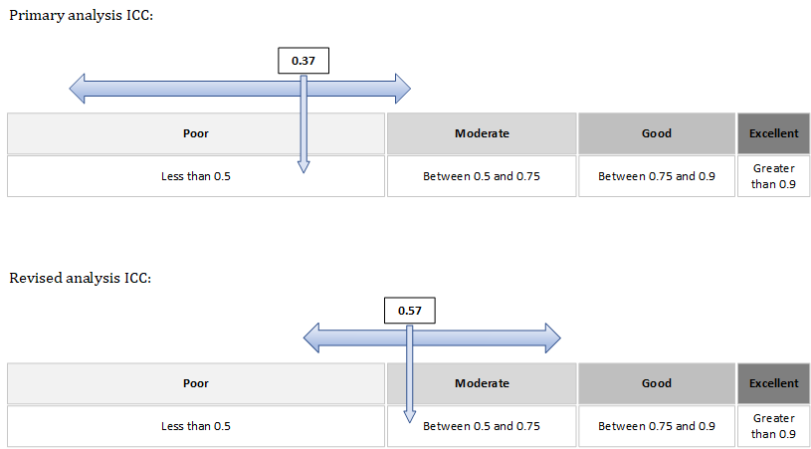
Primary and adjusted analysis calculation of ICC for physiotherapist-DART agreement across all participants and associated confidence interval indicating DART reliability. DART: digital assessment routing tool; ICC: intraclass correlation coefficient.

Considering the second and third amendments, it was of interest to examine if the current protocol revisions would alter the level of agreement between the physiotherapist and DART and influence the calculation of the inferiority margin. The data were reanalyzed, and an ICC was calculated. Adverse outcomes were reduced to 1% (1/78) and with level 1 DART overescalations considered acceptable mitigation of clinical risk, agreement increased to 61/78 (78%; 95% CI 47-78; ICC 0.57, 95% CI 0.40-0.70; Figure 6).

## Discussion

### Principal Results

Pilot studies are considered a crucial element of good study design, increasing the likelihood of success of a main trial, and providing valuable insights for other researchers [58]. The study data collection process proved effective for all predefined outcomes and recruitment targets, confirming a full-scale trial would be feasible to deliver. In addition, the pilot provided valuable insight as to the potential trial burden when recruiting at multiple study sites, study duration, and funding requirements. However, previously recognized challenges inherent in evaluating accuracy of digital triage systems [59] also became apparent, necessitating consideration of their impact for a full trial and associated necessary mitigating actions.

First, we encountered the well-documented epistemological challenge of defining the “gold standard” against which to measure outcome agreement accuracy, this being related to high interrater variability and lack of consensus across clinicians [59-61]. An FCP physiotherapist with extensive postgraduate musculoskeletal training and experience was selected as the gold standard comparator for the pilot; however, there was only full panel agreement with their outcome in 43% (6/14) cases reviewed. Even between the expert panel members themselves, the level of agreement was just 57% (8/14). Previous studies of triage systems have yielded a wide range of clinician or system outcome agreement with predominance of “variable and low accuracy” [62]. The authors suggest the following protocol amendments to improve the consistency of the study clinical comparator: (1) review of all cases by the expert panel where there is a disagreement between DART and physiotherapist outcome, (2) providing panel members with an option of “arguably correct” as an outcome, to better reflect the ambiguity inherent in everyday clinical practice, (3) in conjunction with the NHS service clinical teams, refining and clearly documenting the referral criteria for each routing. However, absolute levels of agreement between digital triage systems and clinicians do not reflect a real-world setting, where consideration of acceptable clinical risk versus resource optimization and minimizing demand on emergency department referrals are important considerations in decision making [30]. The NHS clinical team provided valuable input as to what constituted an acceptable level of clinical risk balanced against limited clinical resources, providing a range of appropriateness (ROA). This included confirmation that DART routing to self-management (incorporating safety netting) when the physiotherapist had routed to physiotherapy, was safe, appropriate, did not constitute an adverse outcome, and would release clinicians to manage more complex cases. They also concluded some DART overescalation of outcome (1 level in this study) was necessary to manage clinical risk. Taking these factors into consideration, the revised ICC calculation increased to 78% agreement with no adverse triage decisions that would put patients at risk, together with a high level of patient satisfaction with DART, which was sufficient for the NHS clinical team to conclude DART had the potential to improve their musculoskeletal pathway. There are currently no studies or regulatory guidelines which define an acceptable level of clinician or system agreement, and therefore no benchmark to determine if DART, or indeed any digital triage system, is “good enough” to implement into clinical practice. The purpose of a definitive noninferiority trial is to provide reassurance that DART would provide safe and effective routing, but to achieve this ROA, a noninferiority margin must first be established. The findings from this pilot study will be used as a basis for more formal consensus to provide a definitive definition of safety criteria, ROA, non-inferiority margin [63], and subsequently calculation of the main study sample size. This will be achieved using a context-specific process [64] recruiting service lead musculoskeletal clinicians (physiotherapists and doctors) to agree on what constitutes an ROA, considering operational services requirements in addition to purely clinical agreement.

Second, we recognized the methodological tradeoff of recruiting real-world patients as opposed to using the more established vignette design [30]. Vignettes typically have higher internal validity especially when assessing agreement for clearly defined symptom presentation, and this method was used during early DART development testing. From a trial delivery perspective using vignettes would be simple and more cost-effective. However, we question the external validity of this approach as real-life patients frequently have complex and ambiguous presentations, with potentially more than 1 appropriate outcome option. This was particularly evident in the poor level of agreement for FCP routing of just 18% (2/11), where boundaries between physiotherapy, FCP, and medical (primary physician) routing were dependent on multiple factors associated with more complex patient presentations. We know from our previous DART usability study, that there are numerous social and emotional factors influencing a patient’s interaction with the system, and ultimately their triage outcome [34], not accounted for with the use of vignettes [65]. While accepting the intrinsic challenges associated with using real patients, we are confident this will provide a more accurate assessment of DART safety and effectiveness than using vignettes as a digital triage comparator [62]. Consequently, to improve the accuracy of DART routing we have introduced prognostic indicators of poor clinical outcomes for these more complex cases into the algorithm, together with matched management recommendations.

Third, are the intrinsic ontological limitations of evaluating a rapidly changing and highly contextual mHealth system such as DART [57,59,66]. A key feature of DART is its ability to fit into an existing musculoskeletal referral process without disrupting the existing pathway. While patients with musculoskeletal conditions present with broadly similar conditions across the United Kingdom, local pathways consist of differing referral criteria and condition management options. To route patients effectively, DART routing must be configured to allow for this variation, producing several service-specific DART variants, and potentially reducing the study’s external validity. However, the use of published clinical guidelines and evidence-based practice applicable within the DART algorithm assists in the consistency of routing based on patient symptomology, while service-specific referral criteria are only configured in the final routing recommendation page. This provides consistency of clinical routing across all DART variations while matching the patient to available services. In addition, we have an established method of assessing DART routing performance in a real-world environment using pre- and postimplementation data, which has proven effective across 8 clients and over 7000 DART assessments to date, without clinical incident.

Finally, a key strength of this trial design was the coproduction model of integrated knowledge translation between the NHS clinical team and researchers. [67] This continued throughout the whole research process, not just in the planning stages, so ensuring methodology was relevant to a real-world NHS musculoskeletal pathway and connecting research to practice [39,67]. The benefits of this approach were highlighted during the revised analysis, where collaborative working refined the method of measuring agreement, leading to a revised ICC calculation. This model of working is considered integral to the success of the main trial.

### Limitations

The generalizability of study results must be considered. Primary care contexts are not homogenous, with geographical factors and patient demographics being key variables [68]. While the demographic consistency of the 2 study arms was well balanced, overall, there was a lesser percentage of men and older participants than would normally be found presenting to primary care with a musculoskeletal condition [7,50]. It is possible that patients of working age were unable to attend a clinic appointment within the daytime hours available, with older less digitally literate people self-selecting. Future research should include offering evening and weekend appointments if practical and encouraging patients to seek assistance with completing their DART assessment if required. The highest level of outcome (medical) was not adequately tested with no participants requiring referral to emergency or urgent primary GP. These are infrequent presentations in first-contact musculoskeletal services, and while the higher number of cases presenting within a larger RCT would be likely to test DART in this area, consideration should be made to “seeding” them into the main trial using vignettes delivered by nonclinicians.

### Bias

This study was funded by Optima Health, the developers of DART, and therefore was at risk of bias. The principal investigator is an employee of Optima Health and enrolled in a PhD program at Queen Mary University of London (QMUL). The other 3 research assistants collected data, one of whom was a physiotherapist and 2 were nonclinicians, all of whom were Optima Health employees. To minimize bias, the researcher had no access to data collected either through DART or by the physiotherapist, nor the SUS web-based questionnaire. No researcher, including the principal investigator, had visibility of the full set of data until data collection had been completed. The study FCP physiotherapist was employed by the NHS Trust with additional clinic time required by the study being funded by the NHS musculoskeletal service. The expert panel consisted of senior musculoskeletal clinicians who were not employed in any form, or paid by, Optima Health. To further mitigate bias, participants were excluded if they were employees of Optima Health or QMUL. Participants had not seen or used DART previously and there was no financial reward offered to people to participate in the study.

### Implications for Progression

This trial demonstrated a definitive RCT is feasible and will use the adjusted protocol described in this paper to examine the agreement between a DART assessment and a usual care physiotherapist assessment, using a predefined noninferiority margin as an indicator of safety and effectiveness. The implications of a successful trial would be to support further DART development progressing to deployment within a real-world NHS musculoskeletal service to achieve improved service delivery. In addition, it would provide a proven methodology for other developers of digital triage systems. The key requirement now to allow progression to the main trial is to achieve a consensus for a noninferiority margin, leading to sample size calculation. This will be achieved using a context-specific consensus process.

### Conclusions

Our study highlighted the well-documented complexity of assessing the safety and effectiveness of a digital triage system and the importance of conducting studies in a live clinical environment. We established study validity was enhanced by the recruitment of real-world patients and engagement of NHS service managers and clinicians, in an integrated knowledge translation approach. The physiotherapist-DART agreement of 78%, with no adverse triage decisions and a high level of patient satisfaction, was sufficient for the NHS clinical team to conclude DART had the potential to improve their musculoskeletal pathway. Completion of a context-specific consensus process is recommended which would provide a definitive definition of safety criteria, range of appropriateness, non-inferiority margin, and sample size in preparation for the main study. This pilot demonstrated an adequately powered definitive trial is feasible, which will provide evidence of DART safety and efficacy, ultimately informing potential DART use in a real-world NHS setting.

## Appendix

Multimedia Appendix 1 Patient Information Sheet.

Multimedia Appendix 2 Queen Mary University of London participant Privacy Notice.

Multimedia Appendix 3 Participant consent form.

Multimedia Appendix 4 CONSORT-eHEALTH checklist (V 1.6.1).

## Abbreviations

DART: digital assessment routing tool
CONSORT: Consolidated Standards of Reporting Trials
FCP: first contact practitioner
GP: general practitioner
ICC: intraclass correlation coefficient
NHS: National Health Service
RCT: randomized controlled trial
ROA: range of appropriateness

## Data Availability

The data sets generated and analyzed during this study are not publicly available as they are stored within the digital assessment routing tool (DART) system, but are available from the corresponding author on reasonable request.
