# Supplementary material for: Evaluation of a Musculoskeletal Digital Assessment Routing Tool (DART): Crossover Noninferiority Randomized Pilot Trial
Source: JMIR Form Res. 2024 Jul 30;8:e56715. doi: 10.2196/56715 (PMC11322692; doi:10.2196/56715)
Supplement: Multimedia Appendix 1 [file formative_v8i1e56715_app1.docx]

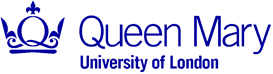


Participant Information Sheet

**Pilot study title:** Validation of a Musculoskeletal Digital Assessment Routing Tool (DART): A Pilot Randomised Crossover Non-Inferiority Trial.

**Running title:**

Does an online digital assessment tool called DART signpost patients as well as a triage physiotherapist for musculoskeletal problems?

**Researcher’s name**

Ms. Cabella Lowe

PhD student

Centre for Sport and Exercise Medicine

Mile End Hospital, London E1 4DG

c.lowe@qmul.ac.uk

**Research Ethics Committee reference number:** 301186

**Invitation**

We would like to invite you to be part of this research project. You should only agree to take part if you wish to do so; it is entirely up to you. If you choose to not take part, there won’t be any disadvantages for you, and you will hear no more about it.

Please take time to read the following information carefully before you decide whether or not to take part. This will tell you why the research is being done and what you will be asked to do should you agree to take part. Please ask if there is anything that is not clear or if you would like more information.

**What is the purpose of the study and what would taking part involve?**

One in five people in the United Kingdom suffers from a problem with their joints, muscles or other soft tissues. For these people, getting help quickly means getting better faster. Some treatments like physiotherapy or a self-help programme can help, but it can take a while to get to the right person who can help you. Some people need to see their GP first or may be asked to see or speak to a physiotherapist before they can get the right help.

Researchers from Queen Mary University London listed at the bottom of this information sheet will be running this study to explore a new way for people to quickly get the care they need using a system called DART (Digital Assessment Routing Tool). DART is an on-line system you can use on a mobile device or home computer. DART asks a series of questions and will be able to sign-post you to the right type of help for your problem. DART has been designed by a company called Optima Health, who are working with Queen Mary University of London to test the safety and effectiveness of the system. The main researcher on this study is employed by Optima Health and is also a PhD student at Queen Mary University London.

If you wish to participate in the study, we will ask you to complete an online assessment of your problem on a tablet device in the GP waiting room, either before or after you have your appointment with the physio for your musculoskeletal problem. You will also be asked to complete a short on-line questionnaire. You will not be given a copy of your DART assessment and it will be the physio who will tell you what treatment you need. You will still see the physiotherapist and have any treatment you need, regardless of whether you take part in the study or not.

This is the process if you take part:

You come to the GP surgery 15 minutes earlier than your physio appointment . The researcher will meet you, answer your questions and get your formal consent to participate

You complete a DART assessment and questionnaire on the tablet device

(15 mins)

You complete an assessment with the physio

(20 mins)

The physio recommends what treatment you should have and you will receive care in the normal way

**Why am I being invited?**

You are being invited to participate in our research study because you are an adult aged 18 years or above who shows signs of a musculoskeletal problem (a problem with a joint, muscle or soft tissue).

**Do I have to take part?**

No, it is up to you to decide whether to take part. If you do wish to consider taking part, you should read this information sheet (which you can keep) and be asked to electronically sign a consent form when you meet the researcher. You can withdraw from the study at any time without needing to provide a reason, and with no disadvantages to your usual care. Your GP will be aware that we are asking patients to participate in this study.

**What are the possible benefits of taking part?**

There is no guarantee that this study will benefit you. However, the information we gather from the trial will help us to test and improve DART, which we hope will allow people to access the right treatment quicker. There will be no changes to your treatment by taking part in this study.

**What are the possible disadvantages and risks of taking part?**

We do not anticipate any disadvantages or risks to you from taking part in this research study. The referral process will take slightly longer than usual (15 minutes) due to the extra DART assessment. We have carefully planned the study to minimise the extra time required. You will still receive your physio assessment on the same day as you would do normally.

**Expenses and payments**

We do not offer any payment or cover expenses for your participation in this study.

**What information about me will you be collecting?**

We will be collecting the responses you have given to the questions in DART and the on-line questionnaire. The information about your musculoskeletal problem and other non-identifiable participant information, such as your age and gender, will be collected and retrieved from DART. We will also ask the physio what sort of treatment they recommended for you. In some cases, we will request the physio assessment record, which will be reviewed by a small panel of clinical experts. They will check to see how well DART agreed with the physio. In these cases, the panel will not know who you are, only your study number will be included. They will only see the record for the assessment you had for your musculoskeletal problem on that day, no other medical information will be shared.

**How will we use information about you?**

We will need to use information from you and your physiotherapist for this research project.

This information will include your name and contact details. People will use this information to do the research or to check your records to make sure that the research is being done properly.

People who do not need to know who you are will not be able to see your name or contact details. Your data will have a code number instead.

We will keep all information about you safe and secure.

Once we have finished the study, we will keep some of the data so we can check the results. We will write our reports in a way that no-one can work out that you took part in the study.

**What are your choices about how your information is used?**

- You can stop being part of the study at any time, without giving a reason, but we will keep information about you that we already have.
- We need to manage your records in specific ways for the research to be reliable. This means that we won’t be able to let you see or change the data we hold about you.

**Where can you find out more about how your information is used?**

You can find out more about how we use your information

- at [www.hra.nhs.uk/information-about-patients/](https://www.hra.nhs.uk/information-about-patients/)
- by asking one of the research team
- by contacting us at Queen Mary’s Data Protection Officer, Queens’ Building, Mile End Road, London, E1 4NS
- by sending an email to [data-protection@qmul.ac.uk](mailto:data-protection@qmul.ac.uk) or
- by ringing us on 020 7882 7596.

You are welcome to ask the study team for the results of the study if you wish.

**Insurance and indemnity**

The insurance that Queen Mary has in place provides cover for the design and management of the study as well as "No Fault Compensation" for participants, which provides an indemnity to participants for negligent and non-negligent harm.

- Insurance and indemnity to meet the potential legal liability of investigators / collaborators arising from harm to participants in the conduct of the research at the GP surgery will be covered by their own professional indemnity and in the case of Optima Health employees, by the cover held by Optima Health.

**What should I do if I have any questions or concerns about this study?**

If you have any questions or concerns about the manner in which the study was conducted please, in the first instance, contact the researcher(s) responsible for the study. If you have a complaint which you feel you cannot discuss with the researchers then you should contact the Research Ethics Facilitators at [research-ethics@qmul.ac.uk](mailto:research-ethics@qmul.ac.uk) or Queen Mary Ethics of Research Committee, Room W104, Queens’ Building, Mile End Campus, Mile End Road, London, E1 4N or Queen Mary Ethics of Research Committee, Joint Research Management Office (JRMO) Empire House, 65 - 75 New Road, Whitechapel, London, E1 1HH. When contacting the Research Ethics Facilitators, please provide details of the name or description the study or QMERC reference number, the researcher(s) involved, and details of the complaint you wish to make.

Contact details

Researcher: Ms. Cabella Lowe

PhD student

Centre for Sport and Exercise Medicine

Mile End Hospital, London E1 4DG

[c.lowe@qmul.ac.uk](mailto:c.lowe@qmul.ac.uk)

07976 315105

Research Supervisor: Professor Dylan Morrissey

Professor of Sport and Musculoskeletal Physiotherapy

Centre for Sport and Exercise Medicine

Mile End Hospital, London E1 4DG

[d.morrissey@qmul.ac.uk](mailto:d.morrissey@qmul.ac.uk)

02082238839
