# Supplementary material for: Evaluation of a Musculoskeletal Digital Assessment Routing Tool (DART): Crossover Noninferiority Randomized Pilot Trial
Source: JMIR Form Res. 2024 Jul 30;8:e56715. doi: 10.2196/56715 (PMC11322692; doi:10.2196/56715)
Supplement: Multimedia Appendix 2 [file formative_v8i1e56715_app2.pdf]

Data Protection Privacy Notice for Research Participants

About this notice

This privacy notice explains generally how Queen Mary University of London (QMUL) processes personal data of individuals who take part in research conducted by us. If you express an interest in taking part in a project or agree to do so, specific information will be made available to you (for example in a participant information sheet) which is supplemented by this notice; the specific information will take precedence should there be any contradiction between these.

QMUL is a data controller in terms of Article 4 of the General Data Protection Regulation and we process personal data in accordance with our Data Protection Policy.

How we use your personal data and the legal basis

You will be informed about the purposes for and the types of personal data used in connection with the specific research you are participating in.

As a publicly-funded university, in general terms, when we use your personal data, we are doing so to conduct research to perform a task in the public interest, as set out in our Charter and Ordinances. If this includes any special category personal data, the legal basis will be that it is necessary for archiving purposes, scientific or historical research purposes or statistical purposes in the public interest.

You are not legally or contractually obliged to supply us with personal data for research purposes.

Sharing your personal data with others

We may have to share your personal data with other parties with whom we are in collaboration. For example, we often work closely with Barts Health NHS Trust and other universities in the U.K. and around the world. We will explain this to all research participants where it applies.

How long your personal data is kept

QMUL retains information in line with its published records retention schedule. You will be advised further about the long-term use (and, where applicable, re-use) and retention of your personal data in connection with the specific research study or project you are participating in.

Your rights and further information

For further information, including on your rights, please see https://www.qmul.ac.uk/privacy/ However, please note that research participants' rights to access, change or move your personal data are limited, as we need to manage your information in specific ways in order for the research to be reliable and accurate. If you withdraw from the study, we will keep the information about you that we have already obtained. Although it is not usually the case with the vast majority of the research we conduct, where we are processing personal data based on your consent, you have the right to withdraw that consent at any time.

Please contact the Data Protection Officer if you have any questions. Other information, including link to a glossary, can be found at: https://arcs.qmul.ac.uk/governance/information-governance/data-protection/
