# Supplementary material for: Evaluation of a Musculoskeletal Digital Assessment Routing Tool (DART): Crossover Noninferiority Randomized Pilot Trial
Source: JMIR Form Res. 2024 Jul 30;8:e56715. doi: 10.2196/56715 (PMC11322692; doi:10.2196/56715)
Supplement: Multimedia Appendix 3 [file formative_v8i1e56715_app3.docx]

**
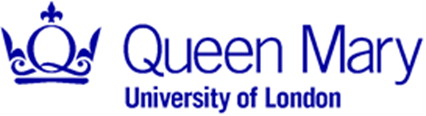
**

Consent Form

**Running Title of Research Study:** Digital Assessment Routing Tool (DART) - Pilot study

**Chief Investigator: Principal Investigator:**

Ms. Cabella Lowe

PhD student

Centre for Sport and Exercise Medicine

Mile End Hospital, London E1 4DG

c.lowe@qmul.ac.uk

Dr Dylan Morrissey

Professor of Sports and Musculoskeletal Physiotherapy

Centre for Sport and Exercise Medicine

Mile End Hospital, London E1 4DG

[d.morrissey@qmul.ac.uk](mailto:d.morrissey@qmul.ac.uk)

**Research Ethics Committee Ref:** 301186

Thank you for your interest in this research.

Should you wish to participate in the study, please consider the following statements. Before signing the consent form, you should initial all or any of the statements that you agree with. Your signature confirms that you are willing to participate in this research, however you are reminded that you are free to withdraw your participation at any time.

| **Statement** | **Please initial box** |
| --- | --- |
| 1. I confirm that I have read the Participant Information Sheet dated 21/07/2021 for the above study; or it has been read to me. I have had the opportunity to consider the information, ask questions and have had these answered satisfactorily. |  |
| 2. I understand that my participation is voluntary and that I am free to stop taking part in the study at any time without giving any reason and without my rights being affected. |  |
| 3. I understand that my data will be accessed by the Chief Investigator and Principal Investigator, Dr Dylan Morrissey and Ms Cabella Lowe respectively. |  |
| 4. I understand that my data will be securely stored on the central server of Queen Mary University of London and in accordance with the data protection guidelines of the Queen Mary University of London for the duration of the study in a de-identified form. Following completion of the study, my data will be stored in an anonymised form. |  |
| 5. I understand that I can request withdrawal and destruction of the information I have provided at any time prior to anonymisation. I understand that following anonymisation, I will not be able to request withdrawal of the personal information I have provided. |  |
| 11. I understand that the researcher will not identify me in any publications and other study outputs using personal information obtained from this study. |  |
| 12. I understand that the information collected about me will be used to support other research in the future, and it may be shared in anonymised form with other researchers. |  |
| 13. I am aware that the advice generated by DART is only for study-related purposes and does not affect or decide the management for your health-related problem. |  |
| 14. I give permission to my treating physiotherapist to share information I give to them as part of the DART study with members of the research team, as described in statement 3, for study-related purposes only. |  |
| 15. I agree to take part in the above study. |  |

Participants should read [Queen Mary’s privacy notice](http://www.arcs.qmul.ac.uk/media/arcs/policyzone/Privacy-Notice-for-Research-Participants.pdf) for research participants which contains important information about your personal data and your rights in this respect. If you have any questions relating to data protection, please contact Data Protection Officer, Queens’ Building, Mile End Road, London, E1 4NS or [data-protection@qmul.ac.uk](mailto:data-protection@qmul.ac.uk) or 020 7882 7596.

__________________ _____________ _____________________________

Participant name Date Signature

__________________ _____________ _____________________________

Researcher Date Signature

I ___________________________________________ confirm that I have carefully explained the nature, demands and any foreseeable risks (where applicable) of the proposed research to the participant and provided a copy of this form.

Participant ID code:

**Chief Investigator**

Professor Dylan Morrissey, Professor of Sport and Musculoskeletal Physiotherapy

Centre for Sport and Exercise Medicine, Mile End Hospital, London E1 4DG

[d.morrissey@qmul.ac.uk](mailto:d.morrissey@qmul.ac.uk)

02082238839
